# Dasatinib as a Bone-Modifying Agent: Anabolic and Anti-Resorptive Effects

**DOI:** 10.1371/journal.pone.0034914

**Published:** 2012-04-23

**Authors:** Antonio Garcia-Gomez, Enrique M. Ocio, Edvan Crusoe, Carlos Santamaria, Pilar Hernández-Campo, Juan F. Blanco, Fermin M. Sanchez-Guijo, Teresa Hernández-Iglesias, Jesús G. Briñón, Rosa M. Fisac-Herrero, Francis Y. Lee, Atanasio Pandiella, Jesús F. San Miguel, Mercedes Garayoa

**Affiliations:** 1 Centro de Investigación del Cáncer, IBMCC, Universidad de Salamanca-CSIC, Salamanca, Spain; 2 Centro en Red de Medicina Regenerativa y Terapia Celular de Castilla y León, Salamanca, Spain; 3 Hospital Universitario de Salamanca-IBSAL, Salamanca, Spain; 4 Departamento de Biología Celular y Patología, Facultad de Medicina, Universidad de Salamanca, Salamanca, Spain; 5 Servicio de Hematología, Hospital General de Segovia, Segovia, Spain; 6 Bristol-Myers Squibb Pharmaceutical Research Institute, Princeton, New Jersey, United States of America; Duke University Medical Center, United States of America

## Abstract

**Background:**

Bone loss, in malignant or non-malignant diseases, is caused by increased osteoclast resorption and/or reduced osteoblast bone formation, and is commonly associated with skeletal complications. Thus, there is a need to identify new agents capable of influencing bone remodeling. We aimed to further pre-clinically evaluate the effects of dasatinib (BMS-354825), a multitargeted tyrosine kinase inhibitor, on osteoblast and osteoclast differentiation and function.

**Methods:**

For studies on osteoblasts, primary human bone marrow mensenchymal stem cells (hMSCs) together with the hMSC-TERT and the MG-63 cell lines were employed. Osteoclasts were generated from peripheral blood mononuclear cells (PBMC) of healthy volunteers. Skeletally-immature CD1 mice were used in the *in vivo* model.

**Results:**

Dasatinib inhibited the platelet derived growth factor receptor-β (PDGFR-β), c-Src and c-Kit phosphorylation in hMSC-TERT and MG-63 cell lines, which was associated with decreased cell proliferation and activation of canonical Wnt signaling. Treatment of MSCs from healthy donors, but also from multiple myeloma patients with low doses of dasatinib (2–5 nM), promoted its osteogenic differentiation and matrix mineralization. The bone anabolic effect of dasatinib was also observed *in vivo* by targeting endogenous osteoprogenitors, as assessed by elevated serum levels of bone formation markers, and increased trabecular microarchitecture and number of osteoblast-like cells. By *in vitro* exposure of hemopoietic progenitors to a similar range of dasatinib concentrations (1–2 nM), novel biological sequelae relative to inhibition of osteoclast formation and resorptive function were identified, including F-actin ring disruption, reduced levels of c-Fos and of nuclear factor of activated T cells 1 (NFATc1) in the nucleus, together with lowered cathepsin K, αVβ3 integrin and CCR1 expression.

**Conclusions:**

Low dasatinib concentrations show convergent bone anabolic and reduced bone resorption effects, which suggests its potential use for the treatment of bone diseases such as osteoporosis, osteolytic bone metastasis and myeloma bone disease.

## Introduction

Bone mass is regulated by the balance of bone formation and bone resorptive rates. Alteration of this balance by increased number and activity of bone-resorbing osteoclasts (OCs) and/or reduced differentiation and impaired activity of bone-forming osteoblasts (OBs), leads to pathological states of bone loss. That is the case of bone diseases such as osteoporosis, osteolytic bone metastasis and multiple myeloma bone disease. In osteoporosis, a prevalent disease of postmenopausal women and elderly patients, bone resorption exceeds that of bone formation resulting in a systemic impairment of bone mass, strength, and microarchitecture [1]. This highly increases the propensity of fragility fractures, most commonly occurring in the spine, hip or wrist. Also, many solid tumors (prostate, breast, lung, colon, renal), commonly metastasize to bone. When this occurs, tumor cells mobilize cellular and extracellular matrix bone components to ultimately promote bone invasion and enhance tumor growth, which leads to deregulated bone remodeling and as a consequence, devastating skeletal complications [2], [3]. Furthermore, multiple myeloma (MM) is a hematological malignancy primarily developing within the bone marrow as a consequence of the abnormal expansion of clonal plasma cells. Interestingly, one major (in up to 80% of MM patients) clinical symptom associated with this disease is the development of osteolytic lesions as a result of increased bone resorption and marked impairment of bone formation. The interactions of myelomatous cells with the bone marrow microenvironment are thought to be critical in the development of MM bone disease, and the diverse interplaying cellular and molecular components have been extensively studied recently [4]–[6]. Of interest, isolated mesenchymal osteoprogenitor cells from the bone marrow of myeloma patients have been reported to show distinct gene expression profile and also reduced osteogenic potential as compared to those from healthy donors [7], [8]. All these low bone mass pathologies (osteoporosis, osteolytic bone metastasis and myeloma bone disease) cause skeletal fragility and are commonly associated to skeletal related events including pathological fractures, severe bone pain, hypercalcemia and spinal cord and nerve compression. These events can severely compromise the quality of life of patients and even result in significant mobidity and increased risk of death. This emphasizes the need to identify and develop new bone-targeted pharmacological agents which may prevent, reduce or even reverse these pathological conditions of bone loss in the above mentioned diseases.

Specific tyrosine kinases have been proposed as potential targets for anti-tumor therapy. Imatinib mesylate (STI-571) is a tyrosine kinase inhibitor which was originally approved as a first-line treatment for chronic myeloid leukemia because of its capacity to inhibit the Bcr-Abl kinase activity of Philadelphia^+^ cells [9]. Additional tyrosine kinases with oncogenic potential also inhibited by imatinib include c-Kit, the platelet-derived growth factor receptors: PDGFR-α and PDGFR-β, and the c-Fms receptor [9], [10], which account for the anti-tumor effect of imatinib in several types of solid tumors. Interestingly, evidence has accumulated for a direct effect of imatinib in the skeleton with increased trabecular bone volume and bone mineral density in imatinib-treated patients [11], [12]. In *vitro* studies showed that imatinib suppressed OB proliferation and stimulated osteogenic gene expression and mineralization majorly by inhibiting PDGFR function [11], [13]. Moreover, imatinib has a potent inhibitory effect on OC bone resorption and stimulates apoptosis of mature OCs [14].

Dasatinib (BMS-354825) is a novel oral bioactive multitargeted tyrosine kinase inhibitor which was developed as a second-generation drug rationally designed for the use against imatinib-resistant leukemias [15]. The target tyrosine kinase profile of dasatinib partially overlaps that of imatinib but presenting much higher potency, and is also broader, including the Src family kinases [10]. Dasatinib is now being evaluated in Phase II trials in a variety of tumor types, including prostate, breast, colorectal and lung cancer [16]. However, taking into account the aforementioned skeletal effects of imatinib, it was expected that dasatinib might be even more effective in inhibiting osteoclastogenesis and promoting bone formation. In fact, it has already been reported that dasatinib inhibits OC formation and resorption capacity, mainly by its potent inhibition of c-Fms on OC progenitors [17], [18]. Also, recent data of dasatinib effect enhancing osteoblastogenesis from mesenchymal progenitors have been reported [19]-[21]; other authors, however, have claimed an inhibitory effect on OB differentiation for this agent in similar settings [22]. In the present study we provide *in vitro* evidences of the effect of low dasatinib concentrations in enhancing differentiation and function of mesenchymal osteoprogenitors from both healthy donors, and interestingly, also from myeloma patients. This anabolic bone effect of dasatinib was also observed in the *in vivo* setting after administration of relatively low dasatinib doses to skeletally-immature mice to avoid the inhibitory effects of the agent on OCs and OC precursors and thus targeting endogenous osteoprogenitor cells. Besides, within the same low nanomolar range of dasatinib concentrations, we show *in vitro* data of additional mechanisms of dasatinib inhibitory effect on OC differentiation [diminished expression of c-Fos and reduced levels of nuclear factor of activated T cells 1 (NFATc1) in the nuclear compartment], and on OC function (F-actin ring disruption and lowered αVβ3 integrin, CCR1 and cathepsin K expression). Taken together, our data support the overall bone anabolic effects of dasatinib, with a double component of enhancement of OB differentiation and function together with inhibition of osteoclastogenesis and bone resorption, exerted within a similar concentration range. Potential therapeutic implications of dasatinib for the treatment of specific bone disorders are also discussed.

## Methods

### Participants

Samples from the bone marrow of 10 healthy donors and 10 newly diagnosed MM patients (stages I to III) were used in this study after informed and written consent of participants. Approval of the study was granted by the Institutional Review Board of the CIC, IBMCC (University of Salamanca-CSIC, Spain), and research was conducted following principles in the Declaration of Helsinki.

### Reagents and immunochemicals

Dasatinib was provided by Bristol-Myers Squibb Company (Stamford, CT, USA). For *in vitro* assays, dasatinib was reconstituted in dimethyl sulfoxide (DMSO; Merck, Hohenbrunn, Germany) at a stock concentration of 100 mM and stored at −20°C; further dilutions were made in tissue culture medium at the time of use. Recombinant human PDGF-BB, macrophage colony-stimulating factor (M-CSF) and receptor activator of NF-κB ligand (RANKL) were purchased from Peprotech (London, UK), while stem cell factor (SCF) was obtained from Strathmann (Hamburg, Germany). Primary antibodies for immunoblotting, immunohistochemical and flow cytometry analyses were directed against: PDGFR-β, phospho-PDGFR-β (Tyr857), Erk1/2, phospho-Erk1/2 (Thr202/Tyr204), NFATc1, histone H1 and cathepsin K, purchased from Santa Cruz Biotechnology (Santa Cruz, CA, USA); phospho-c-Fms (Tyr723), phospho-c-Kit (Tyr719), c-Src, phospho-Src (Tyr416), p38 MAPK, phospho-p38 MAPK (Thr180/Tyr182), Akt, phospho-Akt (Ser473), phospho-β-catenin (Thr41/Ser45), PU.1 and c-Fos, from Cell Signaling Technology (Danvers, MA, USA); CD51/61 and CD191, from R&D Systems (Minneapolis, MN, USA); c-Kit and nucleoporin p62, from BD Biosciences (Bedford, MA, USA); α-tubulin, from Calbiochem (Darmstadt, Germany); dephospho-β-catenin (aa 35–50), from Enzo Life Sciences (Plymouth Meeting, PA, USA), and T-cell factor 4 (Tcf4), from Upstate (Millipore, Billerica, MA, USA). All cell culture media and reagents were purchased from Gibco (Paisley, UK). Trypan Blue Solution 0.4% was delivered by Sigma-Aldrich (St. Louis, MO, USA), and the alamarBlue reagent by Invitrogen (Carlsbad, CA, USA).

### Cell lines

The human mesenchymal stem cell (MSC) line immortalized by expression of the telomerase reverse transcriptase gene (hMSC-TERT) was a generous gift from Dr D Campana (Department of Oncology and Pathology, St Jude Children's Research Hospital, Memphis, TN, USA) [23]. The human osteosarcoma cell line MG-63 was obtained from the American Type Culture Collection (CRL-1427; LGC Promochem, London, UK), and used as an osteoblast-like cell line [24]. Cell lines were grown in RPMI 1640 medium (hMSC-TERT) or DMEM medium (MG-63) supplemented with 10% heat-inactivated fetal bovine serum (FBS), 100 U/mL penicillin and 100 μg/mL streptomycin. All cell types were cultured at 37°C in a humidified atmosphere in the presence of 5% CO_2_–95% air.

### Primary mesenchymal stem cells and osteoprogenitor cells

Primary MSCs from BM samples of healthy donors (n = 10) and MM patients (n = 10) were generated as described by Garayoa et al. [25]. Briefly, mononuclear cells from bone marrow samples were isolated using Ficoll-Paque density gradient centrifugation (ρ 1.073; GE Healthcare, Uppsala, Sweden), cultured in DMEM with 10% FBS, 100 U/mL penicillin, 100 μg/mL streptomycin and 2 mM L-glutamine for four days and selected by their adherence to plasticware. The culture medium was replaced twice weekly until MSC cultures were approximately 90% confluent or had been in culture for a maximum of 21 days; at that point, cells were trypsinized (0.05% Trypsin-EDTA) and expanded in a 1∶3 ratio. At passage 3, selected MSCs from both origins were tested to meet definition criteria according to the recommendations of the International Society for Cellular Therapy [26] and experiments were performed.

To induce *ex vivo* differentiation to OBs, the growth medium of MSCs at 80–90% confluence was replaced by an osteogenic differentiation medium consisting of α-MEM supplemented with 10% FBS, 10 mM β-glycerol phosphate, 50 μg/mL ascorbic acid and 10 nM dexamethasone (all additives from Sigma-Aldrich). MSCs were grown in the osteogenic medium for 7 (early stage of OB differentiation), 14 (pre-OB stage) or 21 days (fully differentiated OBs), replacing the medium every 3 or 4 days, in the absence or presence of specified concentrations of dasatinib.

### Cell proliferation and viability assays

To test whether dasatinib affected the growth capacity of the MSC/OB lineage, the hMSC-TERT and MG-63 cell lines were seeded in 6-well plates at 10^4^ cells/cm^2^ or 2.5×10^3^ cells/cm^2^, respectively, and incubated for 7 days in the absence or presence of different dasatinib concentrations. Cells were then trypsinized (0.05% Trypsin-EDTA) and counted using a Trypan Blue solution and a haemocytometer. The alamarBlue reagent was used to examine cell viability of the hMSC-TERT and primary MSCs from myeloma patients at different time points and dasatinib concentrations along the osteogenic differentiation process, as by manufactureŕs instructions.

In addition, to check whether changes in the number of viable cells were due to diminished proliferative capacity or apoptotic effects of the drug, the hMSC-TERT cell line was stained with PKH67 (Sigma-Aldrich), a green fluorescent cell tracker that is retained in the cell membrane and thus can be used for monitoring proliferation based on dye dilution with each cell division. After PKH67 labeling, cells were seeded in 6-well plates at 10^4^ cells/cm^2^ and incubated for 7 days in the osteogenic differentiation medium in the presence or absence of dasatinib. At the end of the culture period, cells were trypsinized and incubated with phycoerythrin (PE) conjugated Annexin-V and 7-amino-actinomycin D (7-AAD) [Becton Dickinson (BD) Biosciences, Bedford, MA, USA] for complementary apoptosis/necrosis information. The cells were acquired using a FACSCalibur flow cytometer, and data were analyzed using the ModFit program to determine the number of cell divisions and the percentage of cells in each division (compared with undivided colcemid-treated cultures) or the Paint-A-Gate program for percentages of apoptotic cells (BD Biosciences).

### Western blotting analyses

Protein lysates were generated and western blotting procedures were performed as previously described [27]. For subcellular fractionation of proteomic samples, the Qproteome Cell Compartment kit was used (Qiagen GmbH, Hilden, Germany).

Detection of PDGFR-β, phospho-PDGFR-β (Tyr857), c-Kit, phospho-c-Kit (Tyr719), c-Src, phospho-Src (Tyr416), Erk1/2, phospho-Erk1/2 (Thr202/Tyr204), p38 MAPK, phospho-p38 MAPK (Thr180/Tyr182), Akt, phospho-Akt (Ser473), phospho-c-Fms (Tyr723), PU.1, NFATc1, c-Fos, cathepsin K, phospho-β-catenin (Thr41/Ser45), dephospho-β-catenin (aa 35–50), histone H1 and α-tubulin was performed by a standard procedure, using primary and appropriate horseradish peroxidase-conjugated secondary antibodies and a luminol detection system with *p*-iodophenol enhancement for chemiluminescence.

To analyze the effect of dasatinib on PDGFR-β, c-Kit and c-Src tyrosine kinases, the hMSC-TERT and MG-63 cell lines were first incubated with different concentrations of dasatinib for 6 hours and then treated with 10 ng/mL PDGF-BB or 50 nM SCF for 20 minutes prior to protein isolation. To test the effect of dasatinib on c-Fms, c-Kit and c-Src, OC progenitors were incubated with dasatinib for 2 hours and then treated with 50 ng/mL M-CSF or 50 nM SCF for 20 minutes prior to protein isolation.

### Alkaline phosphatase and Runx2 activities and mineralization assay

Primary MSCs were cultured in 12-well plates in MSC medium until reaching ∼80% confluency. Cells were then changed to the osteogenic differentiation medium in the presence or absence of dasatinib (2 or 5 nM) for 7 or 21 days, at which times the alkaline phosphatase (ALP) activity, or the Runx2 activity and mineralization assays were performed.

To measure ALP activity, cells were washed in phosphate-buffered saline (PBS), lysed in ice-cold lysis buffer and protein content determined using the Micro BCA assay kit (Pierce, Rockford, IL). ALP activity was determined by specific hydrolysis of *p*-nitrophenylphosphate into *p*-nitrophenol (Sigma-Aldrich) and quantified by OD reading at 405 nm in triplicate using a microplate reader (Asys UVM340, Biochrom, Eugendorf, Austria). Values were referred to the total protein content of the sample.

When determining Runx2 activity, protein nuclear extracts were prepared using the Qproteome Cell Compartment kit. Quantification of Runx2 activation was performed with the ELISA based Trans-AM (AML-3/Runx2) kit as per manufacturer instructions.

For quantitative analysis of alizarin red staining (ARS), we used the method described by Gregory et al. [28]. Briefly, cells were fixed with 10% ice-cold phosphate-buffered formaldehyde for 10 minutes, rinsed with distilled water and stained with 40 mM alizarin red (pH 4.2) for 20 minutes at room temperature. After several washes to reduce non-specific ARS, stained cultures were photographed with an Olympus DP70 camera on an Olympus 31 inverted microscope. Dye was extracted by acetic acid incubation and sample heating, and measured in triplicate at 405 nm in 96-well plates.

### Real-time RT-PCR analysis

To evaluate the effect of dasatinib on the expression of bone formation markers throughout their osteogenic differentiation, MSCs from MM patients or healthy donors were cultured for 7 or 14 days in the osteogenic differentiation medium in the presence or absence of the drug. Total RNA was isolated using the Rneasy Mini kit (Qiagen GmbH, Hilden, Germany). Reverse transcription was performed with 1.0 μg RNA in the presence of random hexamers and 100 U of SuperScript RNase H reverse transcriptase (Invitrogen). For PCR reactions we used the Step One Plus Real-Time PCR System and TaqMan Gene Expression Assays (Applied Biosystems, Foster City, CA, USA) according to manufacturer's instructions. Assay IDs were: *ALP*, Hs00758162_m1; *COL1A1*, Hs01076777_m1, *Osterix*, Hs00541729_m1, and *Runx2*, Hs01047976_m1. Experiments were performed in duplicate for both the target and the endogenous gene (GAPDH) used for normalization. Relative quantification of the target gene expression was calculated by the comparative threshold cycle (Ct) method: 2^–ΔΔCt^ where ΔCt = Ct _target gene_ – Ct _GAPDH_ and ΔΔCt = ΔCt _dasatinib-treated samples_ – ΔCt _samples in absence of dasatinib._

### In vivo model

For *in vivo* studies, dasatinib powder was dissolved in sterile 80 mM citric acid pH 2.1 to make a 10 mg/mL stock solution and then further dilutions were made in 80 mM sodium citrate pH 3.1. Thirty 5-week-old female CD1 healthy mice were housed at our Animal Care Facility. At this age, healthy CD1 mice are skeletally immature and show very active bone formation and minimal bone resorption, and this model was chosen so that the effect of dasatinib on bone could be majorly ascribed to its action on OBs and not to inhibition of OC formation and function. Animals were divided into three groups (n = 10 in each group) receiving: a) 80 mM sodium citrate pH 3.1 as vehicle; b) a lower dasatinib dose of 2.5 mg/kg, and c) a higher dasatinib dose of 10 mg/kg. Treatment was administered by oral gavage in 0.01 mL volume, in a BID regimen (twice a day), 5 days/week in an attempt to maintain the dasatinib concentration range throughout the day. Serum samples were collected at the beginning of the experiment, and also after 3 and 7 weeks of dasatinib treatment (n = 7 per group). Three to 5 animals per group were sacrificed after 3 and 7 weeks of treatment, and both femurs were dissected for microtomographic imaging (micro-CT), histological and immunohistochemical analyses. All animal experiments were conducted according to Institutional Guidelines for the Use of Laboratory Animals of the University of Salamanca, after acquiring permission from the local Ethical Committee for Animal Experimentation, and in accordance with current Spanish laws on animal experimentation.

Osteocalcin and ALP (as markers of new bone formation) as well as TRAP5b levels (the active isoform of TRAP, specifically expressed by OCs, and thus reflecting OC number and activity), were quantified in collected sera. Markers of bone metabolism were measured by dedicated ELISAs according to manufacturerś guidelines [Alkaline phosphatase (Sigma-Aldrich), Mouse osteocalcin (Biomedical Technologies, Stoughton, MA, USA) and Mouse TRAP Assay (Immunodiagnostic Systems, Boldon, UK)]. For statistical analysis, values for a determined serum marker (e. g. osteocalcin) at each point (control, 3 and 7 weeks of treatment) were normalized for every individual animal to its own osteocalcin level at the beginning of the experiment, and plotted as fold change.

To assess bone morphology and microarchitecture, 10% formalin-fixed femurs were analyzed by a micro-CT system (MicroCATII; Siemens, Knoxville, TN, USA) at 75.0 kVp and 250.0 uA. Seven hundred X Ray projections were acquired during a 200° rotation around the sample, with 1250 ms camera exposure time per projection at full resolution (512×512). The reconstruction of the 3D image was done using COBRA V6.1.8 (Exxim Computing Corporation, Pleasanton, CA, USA) with a final resolution of 10.4 µm/voxel. The post-processing, rendering and generation of the cross sections of the samples was done using Amira (Visage Imaging, San Diego, CA, USA). Analysis of microarchitectural trabecular bone morphology in the distal femur was performed using CT-Analyser software (SkyScan, Kontich, Belgium). Quantitative bone determined parameters were the bone perimeter per area ratio (B Pm/B Ar; mm^−1^), trabecular number (Tb N; mm^−1^) and trabecular separation (Tb Sp; mm).

In parallel, other femurs were also processed for histologic and/or immunohistochemical studies following standard procedures. Briefly, specimens were fixed in 10% formalin for 24 h, decalcified in Osteosoft bone decalcifying solution (Merck KGaA, Darmstadt, Germany) for 5 days and embedded in paraffin. Samples were cut into 3-µm-thick sections and stained with H&E for bone histologic evaluation or either used for immunohistochemical studies. In the latter case, antigen retrieval was carried out in a Pascal pressure chamber (Dako, Glostrup, Denmark) at 90°C for 20 minutes using a Tris-EDTA buffer pH 9.0, and then tissue endogenous peroxidase activity was quenched with a 3% H_2_O_2_ solution for 10 minutes. Sections were incubated overnight with an anti-Tcf4 antibody at 4°C and 1∶20 working dilution, followed by incubation with EnVision anti-mouse complexes (Dako). The peroxidase activity was shown using 3,3′-diaminobenzidine+ (DAB+; Dako) as a chromogen. Finally, sections were washed in water, lightly counterstained with hematoxylin, dehydrated and mounted in DPX. Histologic and immunostained sections were observed with an Olympus BX51 microscope and photographed with a Olympus DP70 camera. Tcf4 is an activating transcription factor which cooperatively interacts with Runx2/Cbfa1 to stimulate osteoblast-specific osteocalcin expression [29], and thus can be used as a bona fide marker for OB cells.

### Osteoclast differentiation and pit formation assays

Peripheral blood monocuclear cells (PBMCs) from 6 healthy donors were obtained by density gradient centrifugation using Ficoll-Paque (ρ 1.073; GE Healthcare), and cultured overnight at 0.5×10^6^ cells/cm^2^ in α-MEM medium supplemented with 10% FBS and 100 U/mL penicillin and 100 µg/mL streptomycin. After removal of non-adherent cells, the remainder were maintained in the same medium but with additional 50 ng/mL RANKL and 25 ng/mL M-CSF in the absence or presence of dasatinib at indicated times and doses. The medium was replaced twice weekly and cultures under each condition were performed in quadruplicate from PBMCs of at least three different donors.

To evaluate the effect of dasatinib on OC formation, cells were stained for tartrate-resistant acid phosphate (TRAP; leukocyte acid phosphatase kit, Sigma), according to the manufacturer's instructions after 21 days of culture. Using a Leica DMI6000 B inverted microscope, TRAP+ cells containing three or more nuclei were enumerated with the aid of a 1×1 mm^2^ grid (five randomly selected fields/well) and photographed with a Leica DFC350FX camera.

To test the effect of dasatinib on the bone resorption capacity of OCs, PBMCs at 0.6×10^6^ cells/well were seeded on calcium-coated slides (Becton Dickinson) in 200 µl of OC medium supplemented with 5 ng/mL TGF-β1 (Peprotech) and 1 µM dexamethasone for 17 days [30]. The medium was changed twice weekly by semi-depletion in the absence or presence of indicated concentrations of dasatinib. At the end of the assay, cells were removed with a 0.1% Triton X-100 solution and resorption pits were photographed using a Leica DFC350FX camera mounted on a Leica DMI6000 B microscope. The total resorbed area per well was calculated using the Adobe Photoshop histogram function (Adobe Photoshop CS2, 9.0.2). Assays were performed in triplicate for each condition using PBMCs from five healthy volunteers.

### Flow cytometry on osteoclast cells

After 2 weeks of osteoclastogenic differentiation in the absence or presence of dasatinib, pre-OBs were trypsinized (0.25% Trypsin-EDTA) and resuspended in Dulbecco's PBS. Cells were stained with anti-CD51/61-PE (αVβ3 integrin-PE conjugated Mouse IgG_1_) or anti-CD191-APC (CCR1-allophycocyanin conjugated Mouse IgG_2B_) for 15 minutes and subsequently with 7-AAD for 5 minutes. After washes, cells were acquired on a FACSCalibur flow cytometer using the CellQuest program and analyzed with the Infinicyt software 1.3 (Cytognos, Salamanca, Spain). Specific staining for CD51/61 or CD191 was evaluated on 7-AAD^-^ viable cells.

### Actin ring formation assay

PBMCs were seeded at 0.5×10^6^ cells/cm^2^ on cover slips and cultured in the specified osteoclastogenic medium for 12–14 days in the presence or absence of the drug. At this point, pre-OCs were fixed in 2% paraformaldehyde for 20 minutes, permeabilized with 0.1% Triton X-100 and blocked in 5% bovine serum albumin in PBS. Cover slips were then incubated with rhodamine-conjugated phalloidin (1∶200; Invitrogen) for 20 minutes to visualize F-actin, and stained with DAPI to make nuclei evident. After a final rinse, cover slips were mounted with an aqueous mounting medium (Vector Laboratories, Burlingame, CA, USA), and the distribution of the F-actin ring was observed with a Leica DMI6000 B microscope and photographed with the Leica DFC350FX camera. Assays were performed in triplicate for each condition using PBMCs from at least three different healthy volunteers.

### Statistical analysis

Each assay was performed at least three times. Quantitative data were expressed as mean ± SD or SEM, as specified. Statistical comparisons using the non-parametric Mann-Whitney *U*-test were considered statistically significant for values of p<0.05 (SPSS Statistics 15.0, Chicago, IL, USA).

## Results

### Dasatinib inhibits PDGFR-β, c-Kit and c-Src phosphorylation and modulates downstream signaling on osteoprogenitor cells

**Figure 1:**
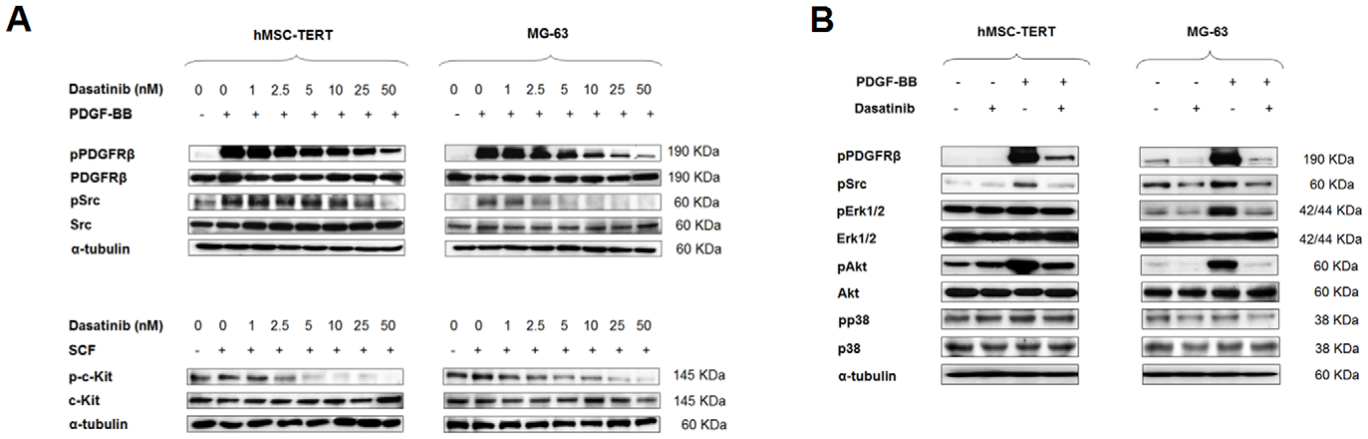
Dasatinib inhibits PDGFR-β, c-Kit and c-Src phosphorylation in mesenchymal and osteoblast-like cell lines. (A) Mesenchymal (hMSC-TERT) and osteoblast-like (MG-63) cell lines were pretreated with different concentrations of dasatinib for 6 hours and then exposed to PDGF-BB or SCF for 20 minutes before protein lysates were generated. Immunoblotting with specific antibodies against total and phosphorylated PDGFR-β, c-Kit and c-Src were performed. (B) Modulation of downstream signaling after dasatinib treatment. Similarly to experimental conditions in (A), the hMSC-TERT and the MG-63 cell lines were pretreated with 50 nM dasatinib for 6 hours, stimulated with PDGF-BB for 20 minutes and then cell harvested for protein isolation. Immunoblotting is shown for total and phosphorylated forms of PDGFR-β, c-Src, Erk 1/2, Akt and p38 mitogen activated protein kinase (MAPK).

We first examined the effect of dasatinib on two tyrosine kinases, PDGFR-β and c-Src, which are described targets for dasatinib and with known involvement in mesenchymal stem cell/osteoblast (MSC/OB) proliferation and function. PDGF is mitogenic to mesenchymal cells while inhibiting OB differentiation and bone matrix formation [31], [32], and targeted deletion of PDGFR-β in murine mesenchymal cells also stimulates OB differentiation and function [33]. On the other hand, knockdown of Src expression has also been shown to enhance OB differentiation and function [19], [34]. Serum-starved hMSC-TERT and MG-63 cell lines (respectively representing the multipotent MSCs and the differentiated OBs), were exposed to PDGF-BB in the absence or presence of escalating doses of dasatinib to check for potential differences in sensitivity due to their differentiation status. As illustrated in Figure 1A, PDGF-BB exposure markedly increased PDGFR-β tyrosine phosphorylation in both cell lines, and dasatinib strongly diminished PDGF-stimulated PDGFR-β activation. PDGF-BB treatment also increased basal p-Y416 of c-Src, ascribable to PDGFR-β activation and its interaction with the Src family kinases [35]. Dasatinib treatment only partially abrogated both basal and PDGF-induced c-Src activation in the hMSC-TERT, while inhibition of c-Src phosphorylation was more pronounced in the MG-63 cell line (Figure 1A). We also show that both cell lines express c-Kit, the stem cell factor (SCF) receptor for which dasatinib also presents inhibitory activity [10], [36]. SCF-induced c-Kit phosphorylation is efficiently diminished by dasatinib in both cell lines (Figure 1A).

To gain some insight into the mechanisms underlying the effect of dasatinib on these cells, we explored the activation status of key molecules in signaling pathways downstream the affected tyrosine kinases, such as: Erk1/2, Akt, and p38 mitogen activated protein kinase (MAPK). As observed in Figure 1B, dasatinib inhibits PDGF-induced Akt activation in the hMSC-TERT and even more effectively in the MG-63 cell line. Inhibition of PDGF-induced PI3-kinase/Akt activation has been shown to promote osteogenic differentiation and mineralized-matrix production in osteogenic cultures [11], [37], and a similar effect could be expected from dasatinib in our experimental settings. On the other hand, dasatinib induced a remarkable inhibition of Erk 1/2 phosphorylation only in the MG-63 cell line and failed to modulate p38 activation in neither cell line. Although activation of Erk 1/2 and p38 have been reported to promote OB differentiation and matrix mineralization through Osterix [38], [39], at least in our experimental settings, dasatinib does not seem to stimulate the osteogenic differentiation and function by similar molecular mechanisms.

### Dasatinib inhibits osteoprogenitor cell and osteoblast proliferation

**Figure 2:**
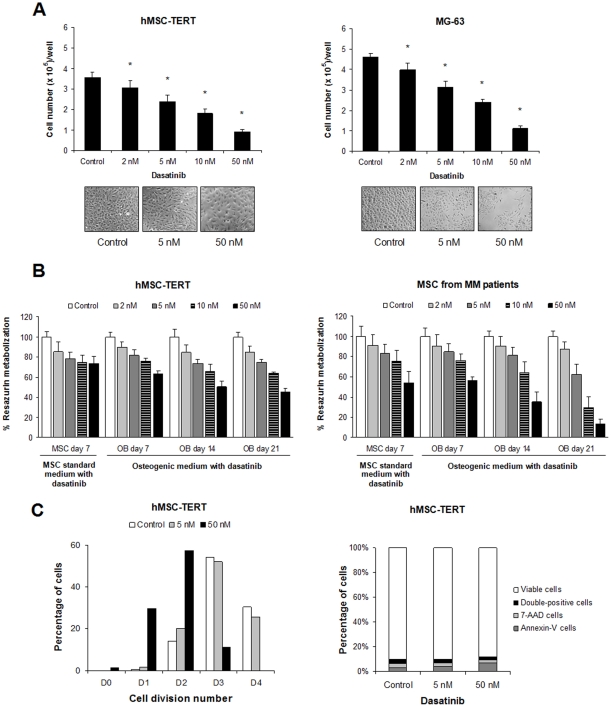
Dasatinib reduces the number of viable cells by inhibition of mesenchymal and OB cell proliferation. (A) Dasatinib decreases the number of MSC and OB cells in culture. The hMSC-TERT and the MG-63 cell lines were cultured for 7 days in maintenance medium in the absence or presence of increasing dasatinib concentrations, and then the number of cells at each condition was counted with a haemocytometer and a Trypan Blue solution. Representative micrographs are shown. (B) Dasatinib reduced the number of viable cells in osteogenic cultures in a time and concentration-dependent manner. MSCs were maintained in osteogenic medium for 7, 14 or 21 days in the presence of different dasatinib concentrations, and percentage of viable cells was evaluated with the alamarBlue assay on OBs derived from the hMSC-TERT (left) and from primary MSCs from MM patients (right). Data are expressed as the mean ± SD from three experiments. Statistically significant differences from control are indicated as *P<0.05. (C) Dasatinib (5–50 nM) reduces the number of cell divisions in the hMSC-TERT cell line (left) but does not induce apoptosis (right). MSCs were stained with PKH67 and cultured in osteogenic medium for 7 days in the absence or presence of dasatinib; at the time of collection, cells were also stained with Annexin-V-PE and 7-AAD and analyzed by flow cytometry.

OBs are derived from precursor MSCs, a process which implies reduction of their proliferative and stem cell self-renewal capacities while acquisition of OB-specific features and lineage commitment [31]. We therefore first tested the effect of dasatinib on the growth rate and viability of OB cells and their mesenchymal progenitors. As seen in Figure 2A, the number of cells in both the hMSC-TERT and MG-63 cultures was progressively reduced with increasing doses of dasatinib. We further studied the effect of increasing dasatinib concentrations on the number of viable cells at different times along the osteogenic differentiation process. A dose and time-dependent reduction on the number of viable cells was observed as referred to the absence of the drug (control) at each time point, which was especially evident at the end of the differentiation period (21 days) and with dasatinib concentrations ≥10 nM (Figure 2B). Also, OBs derived from primary MSCs resulted more sensitive to higher dasatinib doses than OBs derived from the hMSC-TERT cell line (Figure 2B, left graph *vs* right graph), with no significant differences in the number of viable OBs derived from MM patients and healthy donors (data not shown).

It is likely that the reduced number of viable cells in osteogenic cultures after dasatinib treatment could be majorly ascribed to reduced cell proliferation and/or increased apoptosis. Using the hMSC-TERT cell line and a 7-day culture period, we show that dasatinib effect until a 50 nM dose was mainly due to a reduced proliferative capacity (Figure 2C, left) as assessed by reduced number of cell divisions when increasing dasatinib concentration. The percentage of apoptotic and/or necrotic hMSC-TERT cells, however, was only slightly increased within the mentioned dasatinib doses (Figure 2C, right). Our results are therefore consistent with those of other authors which have found that dasatinib inhibits osteoprogenitor cell proliferation [19] and induces apoptosis with higher doses of the drug (≥100 nM) [20].

Importantly, the number of viable cells in OBs derived from primary MSCs (either from MM patients or healthy donors) after the 21-day differentiation period was more pronouncedly diminished as compared to OBs derived from the hMSC-TERT cell line (Figure 2B, left graph *vs* right graph). This issue should be taken into account if dasatinib is used for the treatment of human primary osteoprogenitor cells, in order to achieve a compromise between dasatinib inhibition of cell proliferation and its osteogenic potential. In fact, the use of high dasatinib concentrations might have been the reason why some authors failed to observe an osteogenic effect on human MSCs with this drug [22]. In the light of these observations and for next experiments, we decided to restrict the use of dasatinib concentrations to the low nanomolar range (≤5 nM).

### Low dasatinib concentrations promote osteogenic differentiation, alkaline phosphatase and Runx2/Cbfa1 activities and matrix mineralization

**Figure 3:**
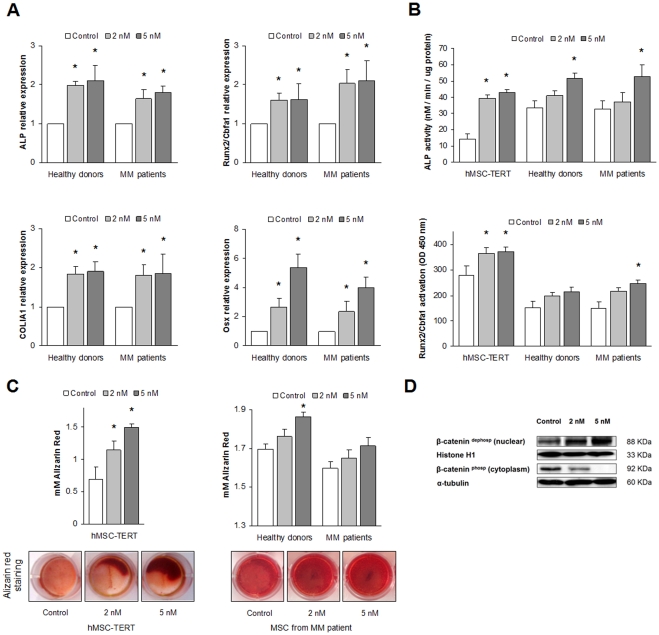
Dasatinib promotes osteogenic differentiation of MSCs from MM patients and healthy donors and of the hMSC-TERT cell line. (A) Dasatinib upregulates the expression of bone-formation markers in the osteogenic differentiation process. Primary MSCs from MM patients and healthy donors were cultured in osteogenic medium in the presence (2–5 nM) or absence of dasatinib, and total RNA was isolated on days 7 and 14. Real-time qRT-PCR was used to determine the expression of several OB related markers: ALP was determined at day 7, whereas the transcription factors Runx2/Cbfa1 and Osterix (Osx), and collagen I type A 1 (COLIA1) were measured at day 14. Expression levels for each gene were normalized to GAPDH expression and referred to those in the absence of dasatinib. Graphs illustrate mean values from samples from 5 healthy donors and 5 MM patients ± SEM (*bars*) *P<0.05. (B) Dasatinib increases ALP and Runx2/Cbfa1 activities in osteoprogenitor cells. In the hMSC-TERT cell line and in primary hMSCs derived from three myeloma patients and three healthy donors, ALP activity was measured at day 7 (upper graph) and Runx2/Cbfa1 activity was measured at day 14 (lower graph) after the addition of dasatinib to the osteogenic differentiation medium. Data are represented as the mean ± SD from three experiments. (C) Dasatinib (2–5 nM) augments bone matrix mineralization in OBs derived from the hTERT-MSC cell line (left) or MSCs from healthy donors and myeloma patients (right), as assessed by alizarin red staining quantification. Data are represented as the mean ± SD from three experiments with the hMSC-TERT cell line, and as the mean (5 MM patients and 5 healthy donors) ± SEM in experiments with primary MSCs. Statistically significant differences from controls are indicated as *, where *P* < 0.05. Micrographs show matrix mineralization after alizarin red staining of correspondent differentiated OBs. (D) Both dephospho- and phospho-β-catenin levels were determined by immunoblotting in cytosolic or nuclear lysates of pre-OBs differentiated from the hMSC-TERT cell line in the absence or presence of dasatinib. Histone H1 and α-tubulin were used as loading controls for nuclear or cytosolic protein fractions.

Since OB maturation implies a balance between proliferation and differentiation, it could be hypothesized that inhibition of osteoprogenitor proliferation by dasatinib would correlate with an enhanced osteogenic differentiation. We evaluated whether dasatinib was capable of modulating osteogenic gene expression in OBs derived from primary MSCs at the selected low concentrations (2–5 nM). Expression of bone-formation markers such as alkaline phosphatase (ALP), collagen I type A 1 (COLIA1) and the transcription factors Runx2/Cbfa1 and Osterix was evaluated by real time RT-PCR and analyzed at either day 7 or 14 of the differentiation process [40]. Figure 3A shows that dasatinib clearly increased the expression of the osteogenic genes to levels higher than those observed in the same conditions but in absence of the drug (control). Interestingly enough, this effect was not restrained to MSCs from healthy volunteers, but MSCs from myeloma patients also responded to dasatinib in a similar way (Figure 3A), thereby supporting the osteoblastogenic therapeutic potential of dasatinib in this disease.

We also examined the biological effect of dasatinib in promoting osteogenic differentiation by measuring ALP and the transcription factor Runx2/Cbfa1 activities as well as mineralized-matrix formation, in OBs differentiated from primary MSCs and from the hMSC-TERT cell line. Dasatinib (at 2 nM or 5 nM) was added to the osteogenic medium at the initiation of OB differentiation, and ALP and Runx2/Cbfa1 activities were measured as surrogates of OB activity at day 7 and 14, respectively. Dasatinib significantly and dose-dependently increased ALP activity even in OBs derived from primary MSCs (Figure 3B, upper). Also Runx2/Cbfa1 activity measured in nuclear lysates of pre-OBs was augmented in the presence of dasatinib, although the increase was not statistically significant in OBs derived from healthy donors (Figure 3B, lower). To estimate matrix mineralization, alizarin red staining and dye quantification were performed after a 21-day osteogenic differentiation of MSCs. Under the aforementioned conditions, OBs derived from the hMSC-TERT cell line underwent a clear dose-dependent increase in mineralization (Figure 3C, left); a slight but reproducible trend towards increased matrix mineralization was also observed in OBs derived from primary MSCs from healthy volunteers and myeloma patients, although it did not reach significance in the latter (Figure 3C, right).

The Wnt/β-catenin signaling pathway is known to play a key role in the osteogenic differentiation of mesenchymal progenitors and in normal skeletal development [41]. Therefore, we examined whether the activity of dasatinib in our experimental setting was accompanied by downstream activation of the canonical Wnt signal transduction. As observed in Figure 3D by immunoblot analysis, dasatinib clearly induced accumulation of the active dephosporylated form of β-catenin in the nuclear compartment, whereas levels of the inactive phosphorylated form in the cytoplasm were reduced. Taken together, our data show that the MSC/OB lineage expresses tyrosine kinases such as PDGFR-β, c-Src, and c-Kit, whose activation can be partially inhibited by low doses of dasatinib (≤5 nM). Within the same range of dasatinib concentrations, these effects are associated with activation of canonical Wnt signaling.

### Dasatinib promotes osteoblast differentiation *in vivo*

**Figure 4:**
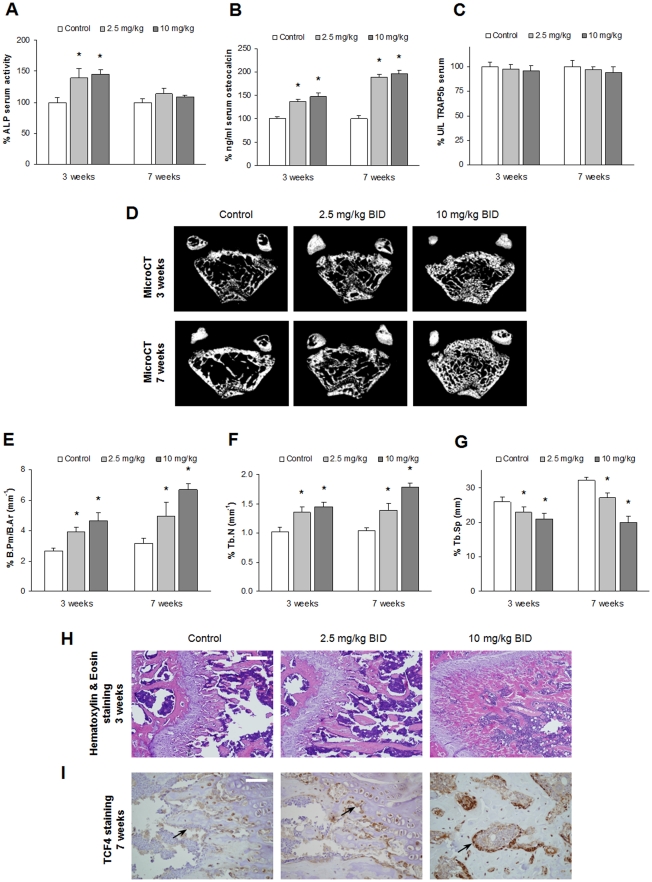
Dasatinib promotes trabecular bone formation *in vivo*. (A, B, C) Five-week-old CD1 mice were treated with vehicle (control) or with dasatinib in a 2.5 mg/kg BID or a 10 mg/kg BID regimen for 3 or 7 weeks, and serum levels were determined for ALP (A), osteocalcin (B) or TRAP5b (C) before initiation of the experiment and at each time point. Graphs are plotted as mean values of fold change from baseline levels for the mentioned factors in sera ± SEM (*bars*). *, *P*<0.05 indicates significant differences between levels for each time and dose of dasatinib and untreated mice at the same conditions (control). (D) Representative micro-CT analyses of equivalent cross-sections of distal femurs are shown for each dasatinib concentration and time of treatment. (E, F, G) Trabecular bone morphometric parameters from micro-CT images were quantitated by CT-Analyser software for bone perimeter per area (E), trabecular number (F) and trabecular separation (G). *, *P*<0.05 relative to vehicle control at each time-point; n = 3 femurs per group. (H) Representative femur sections treated with both dasatinib doses for 3 weeks and stained with hematoxylin and eosin. Bar = 50 µm. (I) Representative images of Tcf4 immunohistochemistry in dasatinib-treated femurs for 7 weeks. OB-like cells immunostained for Tcf4 can be observed lining the trabeculae (arrows). Bar = 12.5 µm.

Consistent with our *in vitro* findings, we corroborated the bone anabolic properties of dasatinib in the *in vivo* setting. To better discern the putative bone formation effect of dasatinib from its known inhibitory activity on OC formation and function, we used skeletally-immature 5 week-old healthy mice which present very active bone formation and minimal bone resorption. Two different doses of dasatinib (2.5 mg/kg *vs* 10 mg/kg) administered twice-a-day (BID) and two periods of dasatinib treatment (3 *vs* 7 weeks) were compared in order to evaluate potential osteogenic/anti-proliferative activities of the drug on endogenous osteoprogenitor cells, as we had observed *in vitro* for primary MSCs. Being ALP an early marker of bone formation, Figure 4A shows that ALP levels in serum were significantly increased in mice treated with both doses of dasatinib after 3 weeks of treatment, whereas ALP levels remained unaffected with respect to vehicle-treated animals at longer treatment periods. Relative to osteocalcin (also a bone formation marker, but expressed at later stages of OB differentiation), significant increases in serum were observed for both doses of dasatinib after 3 weeks and even further increments were attained in a 7-week period. Minimal differences were found in osteocalcin serum levels between the 2.5 mg/kg BID and 10 mg/kg BID doses, neither after 3 weeks nor after 7 weeks of treatment, which probably reflects a near to plateau-effect on osteocalcin induction with the doses in our study. Due to the use of young healthy mice with limited OC function, no changes on levels of TRAP5b (a surrogate marker for OC number) were measured between baseline and after 3 or 7 weeks of treatment (Figure 4C). This is in accordance with a very scarce presence of OCs observed in the histological sections from femurs of control animals along the experiment (data not shown).

The effects of both doses of dasatinib were also evaluated by quantitative micro-CT scanning of distal femurs of treated mice. As observed in Figure 4D, dasatinib treatment led to a marked increase in trabecular microarchitecture of cancellous bone in a dose- and time-dependent manner. This effect was associated to significant increases of trabecular number (Tb N; mm^−1^) and of the ratio of bone perimeter per bone area (B Pm/B Ar; mm^−1^), together with decreased trabecular separation (Tb Sp; mm) compared with vehicle-treated animals (Figures 4E-G). The effects of dasatinib on increased trabecular structures were more pronounced for the 10 mg/kg BID and the 7-week period treatment as compared to the rest of the experimental conditions. The increased trabecular number was equally apparent by histologic observation of newly formed trabeculae at the epiphyseal plate (Figure 4H), and also correlated with increased number and intensity of staining of Tcf4 positive OB-like cells lining the trabecular borders (Figure 4I). The transcriptional activation of target genes by Tcf transcription factors mediates the activation of the canonical Wnt/β-catenin signalling pathway, which is essential in OB differentiation [41]. Specifically, within the Tcf family members, Tcf4 is the one most abundantly expressed in OB cell lines and primary human MSCs [42]; therefore, the increased number and intensity of Tcf4 positive cells may well reflect an increased number of active OBs after dasatinib treatment.

### Dasatinib inhibits osteoclast formation and activity

**Figure 5:**
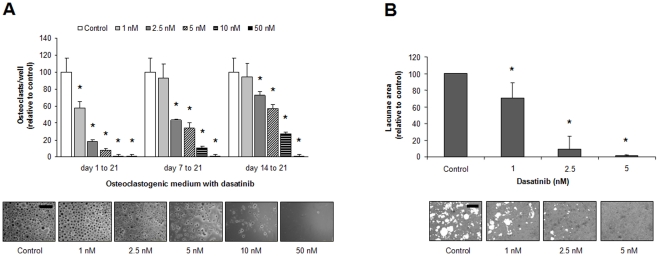
Dasatinib treatment inhibits OC formation and resorption activity. (A) PBMCs from healthy donors were cultured in medium containing M-CSF/RANKL for 21 days in the absence or presence of dasatinib for the indicated times, and OCs were counted (as assessed by TRAP+ staining and the presence of more than three nuclei). Representative micrographs of TRAP staining for OCs treated with dasatinib for 3 weeks are shown. *Bar* =  0 μm. (B) OCs were generated on calcium-coated slides, and the effect of different dasatinib concentrations on OC resorption was evaluated by calculation of the total area of resorbed lacunae. Graphs represent mean values of samples from OCs derived from three healthy donors ± SEM (*bars*). *, *P*<0.05 indicates significant differences between dasatinib-treated cultures and untreated control at the same conditions. Representative micrographs of resorbed lacunae on the calcium-coated wells are shown. *Bar* = 30 μm.

We first confirmed the inhibitory effect of dasatinib in osteoclastogenesis (Figure 5A) and OC function (Figure 5B), as has already been reported for this drug [17], [18]. For this purpose, PBMCs from healthy volunteers were incubated in an M-CSF/RANKL-containing medium for 21 days, and dasatinib was added throughout the differentiation process or on days 7–21 or 14–21. As seen in Figure 5A, when dasatinib was present for 21 days, it markedly reduced OC numbers in a dose-dependent fashion (IC_50_ = 2.16 nM; *P*<0.05 at ≥1 nM *vs.* control). When dasatinib was added to early OC progenitors (day 7) or to committed OC precursors (day 14) it was also effective in reducing osteoclastogenesis, although higher doses were required: IC_50_  = 3.14 nM; *P*<0.05 at ≥2.5 nM *vs* control (7–21 days); IC_50_  = 5.62 nM; *P*<0.05 at ≥ 2.5 nM *vs* control (14–21 days). Notably, the number of OCs was markedly reduced at higher doses of dasatinib (Figure 5A, e.g., ≥5 nM dasatinib, 1–21 days). This could be explained by a toxic effect of dasatinib on OC progenitors at those doses, but it may well also reflect that dasatinib is targeting essential pathways for OC viability.

Figure 5B shows the area of resorptive pits. Progressive substantial reductions of resorbed lacunae were observed with increasing dasatinib concentrations, resorption being almost completely abrogated at a concentration of 2.5 nM. Of note, this effect of dasatinib on OCs is achieved within similar low doses of dasatinib (low nanomolar range) as for its activity in promoting *in vitro* osteogenic differentiation from mesenchymal precursors. Thus, *in vitro* doses of 2–2.5 nM dasatinib on OCs are sufficient for inhibition of OC formation to a 20% of the control and to reduce the resorptive activity further to a 5% of the control, and would not interfere with the osteogenic activity of this compound.

### Mechanism of action of dasatinib on osteoclasts

**Figure 6:**
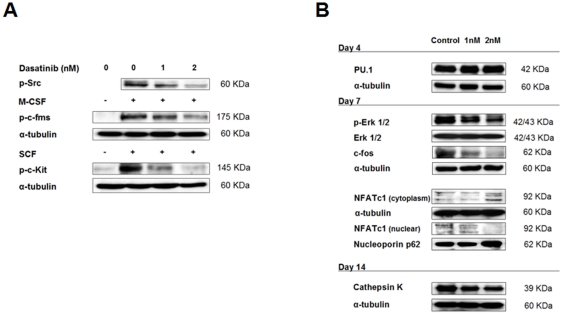
Dasatinib regulates the expression of important molecules/factors for OC formation, differentiation and activity. (A) Dasatinib inhibits c-Fms, c-Src, and c-Kit tyrosine kinase phosphorylation in committed OC precursors. PBMCs were differentiated in osteoclastogenic medium for 7 days, pretreated with 1 nM or 2 nM dasatinib or vehicle, and exposed to 50 ng/mL M-CSF or 50 nM SCF for 20 minutes prior to protein isolation. Immunoblotting with specific antibodies was performed as indicated. (B) PBMCs were maintained in osteoclastogenic medium for indicated times in absence or presence of 1 nM or 2 nM dasatinib. Immunoblots are shown for PU.1, Erk1/2, p-Erk1/2, c-Fos, NFATc1 (both in nuclear and cytoplasmic protein fractions) and cathepsin K.

We first assessed that the low concentrations of dasatinib capable of reducing OC formation and resorption in our previous experiment (1–2 nM) were also effective in inhibiting the activation of the M-CSF receptor, c-Fms, in OCs (as is shown in Figure 6A). Since M-CSF and RANKL are the two main proliferation and survival factors involved in osteoclastogenesis from monocyte/macrophage precursors, the c-Fms kinase has been considered as a major target of dasatinib on OCs [17], [18].

However, since dasatinib is capable of inhibiting OC formation when not present throughout the differentiation process but also at later stages of OC differentiation (days 7–21 and 14–21; Figure 5A), together with the fact that OC resorption is more effectively reduced than OC formation (Figure 5A *vs* 5B), indirectly suggests that inhibition of additional tyrosine kinases other than c-Fms, are also contributing to dasatinib effects on this cell type. Figure 6A shows that in OC precursors, even at doses as low as 1 or 2 nM, dasatinib is capable of inhibiting the activation of two other tyrosine kinases, such as c-Src and c-Kit. The c-Src kinase is an essential molecule for OC resorption, intervening in the αVβ3 integrin outside-in signaling in the sealing zone between the OC and the bone matrix [43]; in accordance with these observations, c-Src ^-/-^ mice show an osteopetrotic phenotype with OCs unable to form ruffled borders despite a normal morphological appearance [44]. Thus, inhibition of this tyrosine kinase by dasatinib would greatly compromise OC functionality. On the other hand, the ligand for c-Kit, the SCF, has been shown to be mitogenic for OC precursors and to promote mature OC activity [45]. Inhibition of signaling through c-Kit by dasatinib may therefore also play a role in inhibition of osteoclastogenesis and diminished OC resorption.

Besides, when analyzing the expression of several key molecules implicated in OC commitment/differentiation/function, we were able to identify further and novel consequences of dasatinib treatment on this cell type. As shown in Figure 6B, in early OC progenitors (day 4 since initiation of *in vitro* differentiation) dasatinib does not affect levels of PU.1, which is a transcription factor that regulates the commitment of myeloid cells to common progenitors for macrophages and OCs [45]. At a later stage of OC differentiation (day 7), dasatinib treatment is associated with a slight inhibition of p-Erk 1/2, and specifically, a marked reduction of c-Fos levels. Notably, c-Fos is a key regulator of OC differentiation and is clearly required for osteoclastogenesis [45], [46]. Mice lacking c-Fos develop osteopetrosis due to defective OC differentiation, whereas the number of macrophages increases [46], [47]. We also show that NFATc1, a major transcription factor integrating RANKL signaling in terminal differentiation of OCs [45], [48] is retained in the cytoplasmic fraction while nuclear NFATc1 levels are diminished after dasatinib treatment for 7 days (Figure 6B). NFATc1 requires dephosphorylation and nuclear translocation to activate the transcription of OC-specific genes [49], and thus the diminished transcriptional activity of NFATc1 would likely contribute to the inhibitory effects of dasatinib in OC differentiation. Besides, in late OC precursors (day 14, prefusion OCs and multinucleated OCs), dasatinib treatment reduces the expression of cathepsin K, which is the major cysteine protease in OCs implicated in degradation of organic cellular matrix during bone resorption [43]; therefore, our data provide another mechanism by which dasatinib may inhibit OC resorption.

**Figure 7:**
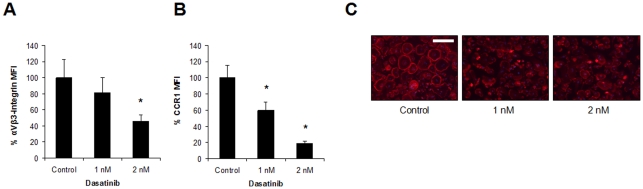
Further consequences of dasatinib treatment on OC function. Expression of αVβ3-integrin (CD51/61) (A) and CCR1 (CD191) (B) was evaluated by flow cytometry in pre-OCs after culture in an osteoclastogenic medium in the absence or presence of dasatinib for 2 weeks. Graphs represent the mean values of the median fluorescence intensity (MFI) percentage from OCs derived from three healthy donors ± SD (*bars*). *, *P*<0.05 indicates significant differences between dasatinib-treated cultures and untreated control. (C) The integrity of the F-actin ring in multinucleated OC precursors (obtained like in A and B) was evaluated by phalloidin-rhodamine staining, whereas nuclei were visualized with DAPI. Representative micrographs for each condition are reported. Bar = 50 µm.

Furthermore, dasatinib treatment on OCs was also associated to a clear reduced expression of the αVβ3 integrin and of CCR1 (Figure 7A, B), and to disruption or even absence of the F-actin ring in most multinucleated OC precursors (Figure 7C). The αVβ3 integrin mediates the interactions between OCs and the extracellular matrix, and is therefore implicated in cell adhesion, regulation of OC migration and bone resorption [43]. The reduced levels of αVβ3 together with inhibition of c-Src activation, would likely account for the disruption of the F-actin ring, which is necessary for the maintenance of the sealing zone and an effective bone resorption [50]. Also, CCR1 is the major receptor for CCL3 (MIP-1α), a pro-inflammatory cytokine that induces osteoclastogenesis and stimulates OC activity [51]–[53]. It is therefore conceivable that downregulation of CCR1 by dasatinib would further sustain dasatinib inhibitory effects in OC formation and resorption. Taken together, we could say that at very low concentrations (1–2 nM) dasatinib is capable of targeting various tyrosine kinases (including c-Fms, c-Src, c-Kit), which by several avenues lead to a profound inhibition of osteoclastogenesis and of OC function.

## Discussion

Mesenchymal stem cells from the bone marrow may under specific conditions differentiate into osteoblasts, adipocytes, chondrocytes, tenocytes, skeletal myocytes and cells of visceral mesoderm [54], [55]. Considerable interest has been raised in recent years for the use of MSCs for repair and regeneration of a number of tissues including bone [56]–[59]. Moreover, the possibility of pharmacologic agents targeting this population of progenitor cells to specifically enhance their differentiation into the osteogenic lineage, further expands their potential as a strategy for bone regenerative medicine.

In concordance with these expectations and also in line with previous data from other groups [19]–[21], we were able to observe that dasatinib treatment effectively promoted the osteogenic differentiation of mesenchymal progenitors (both primary bone marrow MSCs and the hMSC-TERT cell line) as observed by increased ALP and Runx2 activities, augmented matrix mineralization and elevated expression levels of genes associated with OB differentiation (Runx2/Cbfa1, Osterix, ALP and COLIA1). We have also shown that MSCs and OBs express various tyrosine kinases such as PDGFR-β, c-Src and c-Kit, and although with some differences in sensitivity between MSCs or differentiated OBs, dasatinib at low concentrations (≤5 nM) was capable of partially inhibiting their phosphorylation. It is likely, therefore, that concomitant inhibition of these three kinases might be mediating the osteogenic differentiation in our experimental conditions. Other authors have linked the enhanced OB differentiation of dasatinib to its inhibitory activity on the c-Src kinase [19], [20] and on the Abl kinase [19]. We and others have shown that dasatinib promotion of OB differentiation and function relies on inhibition of cell proliferation at lower doses [19] and to induction of apoptosis with higher doses of the drug [20]. Since we observed that primary MSCs (both from normal donors and myeloma patients) are more sensitive to this effect of dasatinib than the hMSC-TERT cell line, it is worth to mention that if dasatinib is used in the clinical setting to pursue an osteogenic effect, special precaution should be taken to achieve a compromise within reduced osteoprogenitor cell numbers and enhanced osteogenic differentiation.

Interestingly, and in support of our *in vitro* observations on the osteogenic promotion activity of dasatinib, these effects were also reflected in our *in vivo* model. Specifically, 5-week-old skeletally-immature mice with very active bone formation and minimal bone resorption were used, so that the effect of dasatinib on bone could be majorly ascribed to its action on OBs and not to inhibition of OC formation and function. Our data showed that both doses of dasatinib were associated with significant increases of trabecular architecture parameters (as calculated from micro-CT analyses) and a higher number of trabeculae on histologic sections of cancellous bone in distal femurs. Although the increased trabecular structures could also result from the inhibitory effect of dasatinib on OC formation and resorption, the augmented serum levels of bone formation markers (ALP, osteocalcin), the increased number and activation of OB-like cells (as observed by Tcf4 immunostaining), together with absence of significant changes in serum TRAP5b levels (as a surrogate for OC number), lead us to conclude that in our model the augmented trabecular formation after dasatinib treatment is majorly attributable to increased OB formation and activity rather than to an inhibitory effect on OCs. It should also be noted that both doses used in our *in vivo* study are relatively low as compared to those used for this drug in mouse models of tumor malignancies, and near the considered minimum efficacious doses of dasatinib (1.25 mg/kg BID or 2.5 mg/kg once a day) [60]. From preliminary studies (data not shown), we know that levels of bone formation markers (ALP, osteocalcin) were not increased as compared to controls in mice treated with a higher dose of dasatinib (25 mg/kg, BID, 5 days/week), which in line with our *in vitro* studies, highlights the importance of maintaining a low and constant concentration of dasatinib to promote the osteogenic differentiation of osteoprogenitors. It should be mentioned that in another model of physiological bone turnover, skeletally-mature 9-month-old rats were treated with a dasatinib dose of 5 mg/kg once a day (thus quantitatively equivalent to our low dose of 2.5 mg/kg on a BID regimen). Serum OB markers were not significantly altered in this study [61], and increases in tibial trabecular bone volume in the rat model were attributed to dasatinib inhibition of OC activity. This discrepancy in both *in vivo* models may be explained by species differences in sensitivity of osteoprogenitor cells to dasatinib, but also likely to differences in experimental models. Consequently with our observations, the capacity of dasatinib to target bone marrow MSCs and to promote their osteogenic differentiation could be used in the biologic repair of skeletal defects of traumatic origin. For instance, dasatinib could be used as an adjuvant therapy to promote endogenous MSC osteogenic differentiation and accelerate bone fracture healing and bone implant fixation. Moreover, dasatinib treatment after establishment of MSC-based bone grafts could improve bone repair and regeneration in the field of orthopaedic surgery.

On the other hand, we were able to confirm the inhibitory effects of dasatinib on osteoclastogenesis and OC resorption *in vitro* [17], [18]. These effects were achieved at very low doses (1–2 nM), and in fact we showed that these concentrations were effective in inhibiting the activation of c-Fms, c-Src and c-Kit (Figure 6A) which are essential tyrosine kinases for OC differentiation and function. When analyzing the expression of several key molecules in the presence of these low dasatinib concentrations, we were able to identify further and novel consequences of dasatinib treatment which would probably contribute to inhibition of OC differentiation (such as reduced c-Fos levels and NFATc1 levels in the nuclear compartment, as well as diminished expression of the CCR1 receptor), and to impair OC resorption (reduced αvβ3 integrin, CCR1 and cathepsin K protease expression, and F-actin ring disruption). Therefore, dasatinib treatment would by several mechanisms lead to a profound inhibition of OC formation and OC function. As previously mentioned, dasatinib inhibitory effect on OCs has also been shown in an *in vivo* model [61].

It is noteworthy to mention that our inhibitory *in vitro* effects of dasatinib on OC formation and function were achieved within the same low nanomolar range (≤5 nM) of concentrations at which dasatinib promoted the *in vitro* osteogenic differentiation from mesenchymal precursors. Besides, those doses have been reported to be safe and therapeutically achievable in pharmacological studies [60], [62]. In our *in vivo* model, we have shown effective bone anabolic effects targeting the osteoprogenitor population also at relatively low dasatinib concentrations (2.5 mg/kg–10 mg/kg) [60]. This likely suggests that there is a therapeutic dosage window of easily pharmacologically achievable low dasatinib concentrations in which concurrent bone formation would be enhanced and bone resorption would be impaired, thus making dasatinib a potential attractive pharmacological approach for the treatment of bone diseases coursing with bone loss and in which both of these processes are affected. In osteoporosis, progressive bone loss results because the osteoblastic activity cannot compensate for excessive bone resorption. Although the standard of care for osteoporosis patients has traditionally relied on anti-resorptive drugs [1], [63], last decade advances in the knowledge of bone biology have highlighted the need for additional anabolic treatments in this disease, and several agents, including calcilytic drugs and antagonists of Wnt inhibitors (such as antibodies against sclerostin and anti-DKK1) are now being evaluated in clinical trials (reviewed in [1]). It can be envisioned that the simultaneous bone-forming and anti-resorptive effects of low doses of dasatinib may well be exploited for the treatment of this disease. Also, in osteolytic-type tumor metastases (most common in metastasis of breast, lung and renal cancer), the enhanced differentiation and resorption activity of OCs, is also accompanied by suppressed OB formation due to DKK-1 secretion from tumor cells [3], [64]. Therefore, convergent anabolic and anti-resorptive activities of dasatinib could be investigated for beneficial impact as an adjuvant treatment besides regular tumor chemotherapy in metastatic skeletal osteolytic lesions.

The potential therapeutic use of dasatinib as an adjuvant therapy in myeloma-associated bone disease deserves a separate comment. The osteolytic lesions in MM are also characterized by augmented OC numbers and resorption and almost suppressed osteoblast OB differentiation and bone formation [5], [65]. The interaction of myeloma cells with stromal and osteoprogenitor cells in the bone marrow leads to the overexpression of multiple OC activating factors (including RANKL, CCL3, IL-3, osteopontin, IL-6 and vascular endothelial growth factor; in turn, OCs also support myeloma proliferation and survival by production of myeloma growth factors such as IL-6, B-cell-activating factor, and a proliferation inducing ligand [4]. The very potent inhibitory effects of dasatinib on osteoclastogenesis and OC function [17], [18] and our own data strongly support that dasatinib would greatly reduce OC numbers and resorption in the context of myeloma bone disease. Besides, we have shown that dasatinib treatment reduces CCR1 expression on late OC precursors (Figure 7B), which is the major receptor for CCL3 (MIP-1α), a crucial stimulator of osteoclastogenesis and of OC function in MM [51], [52]. This would therefore further support an inhibitory resorptive effect of dasatinib in the context of myeloma bone disease. On the other hand, reduced osteoblastogenesis in MM relies on abnormal (genetic, functional and phenotypical) properties and impaired osteogenic potential of osteoprogenitor cells from myeloma patients [7], [25], [66], [67], together with production of multiple osteoblastogenesis inhibitors by myeloma cells and the microenviromental cells within the myelomatous bone [5], [68], [69]. Interestingly, in the present report we have shown that bone marrow MSCs from MM patients, although having a reduced osteogenic capacity [7] are also capable to respond to dasatinib and differentiate to OBs in a similar way as those from normal donors. Preclinical efficacy of dasatinib in multiple myeloma, with specific inhibition of proliferation of myeloma plasma cells and angiogenesis has already been reported [70]. It should be noted, however, that both the *in vitro* stimulatory action of dasatinib on MSCs from myeloma patients as well as its inhibitory effect on OC formation and function are attained at doses in the low nanomolar range (2–5 nM), which are subapoptotic for myeloma cells (IC_50_ = 25–100 nM) and for inhibition of angiogenesis (IC_50_ = 50 nM) [70]. Thererefore, if dasatinib at low doses is to be used in multiple myeloma for a beneficial effect on osteolytic lesions it should be added as a supportive therapy together with other pharmacological agents targeting myeloma growth. Current standard management of MM bone disease relies primarily on bisphosponates (pamidronate, ibandronate, zolendronic acid), which are strong bone-resorption inhibitors but do not stimulate bone formation [71], and may induce adverse side effects such as osteonecrosis of the jaw and renal impairment [72]. Although bisphosphonates are very effective in reducing skeletal complications, bone disease still progresses at a slower rate, which highlights the importance of the clinical incorporation of strategies that may not only impede bone degradation but also promote an anabolic bone effect in multiple myeloma [4], [68]. In line with these treatments, our data strongly suggest that dasatinib at low doses may be a valuable adjuvant therapy for the treatment of myeloma-associated bone disease.

In summary, our results provide evidence that low dasatinib concentrations (2–5 nM) are capable of *in vitro* promoting the osteogenic differentiation and OB activity of primary MSCs, including those derived from MM patients. A bone anabolic effect of dasatinib was also observed in a skeletally-immature mouse model with relatively low doses of dasatinib (2.5 mg/kg BID and 10 mg/kg BID), as assessed by increased trabecular structures, elevated serum levels of bone building markers and higher number of active OBs; since no significant changes were found in sera for TRAP5b (a surrogate marker for the number of OCs), the increased bone trabeculae were ascribed to the promotion of OB differentiation and enhanced activity of endogenous mesenchymal progenitors. In addition, in the same range of low nanomolar concentrations, we showed that dasatinib is capable of hindering *in vitro* osteoclastogenesis and resorption activity and of inhibiting the activation of c-Fms, c-Src and c-Kit kinases. Some of the molecular mechanisms mediating these effects on the OC population have also been identified in this study, including some inhibiting OC differentiation (reduced c-Fos and nuclear NFATc1 levels) and function (reduced expression of αvβ3, CCR1, cathepsin K and F-actin ring disruption). Therefore, our data confirm and provide new insights of dasatinib at low doses as a bone modifying agent with convergent bone anabolic and anti-resorptive effects at therapeutically and safe achievable concentrations. Specifically, dasatinib at low concentrations might be used as an adjuvant therapy to promote the osteogenic differentiation of endogenous or ectopically implanted MSCs. Also, dasatinib holds promise to be therapeutically beneficial for bone disorders coursing with augmented bone resorption and inhibited bone formation, such as osteoporosis, osteolytic tumor metastasis and myeloma bone disease.

## References

[1] Rachner TD, Khosla S, Hofbauer LC (2011). Osteoporosis: now and the future.. Lancet.

[2] Vallet S, Smith MR, Raje N (2010). Novel bone-targeted strategies in oncology.. Clin Cancer Res.

[3] Weilbaecher KN, Guise TA, McCauley LK (2011). Cancer to bone: a fatal attraction.. Nat Rev Cancer.

[4] Basak GW, Srivastava AS, Malhotra R, Carrier E (2009). Multiple myeloma bone marrow niche.. Curr Pharm Biotechnol.

[5] Yaccoby S (2010). Advances in the understanding of myeloma bone disease and tumour growth.. Br J Haematol.

[6] Fowler JA, Edwards CM, Croucher PI (2011). Tumor-host cell interactions in the bone disease of myeloma.. Bone.

[7] Corre J, Mahtouk K, Attal M, Gadelorge M, Huynh A (2007). Bone marrow mesenchymal stem cells are abnormal in multiple myeloma.. Leukemia.

[8] Todoerti K, Lisignoli G, Storti P, Agnelli L, Novara F (2010). Distinct transcriptional profiles characterize bone microenvironment mesenchymal cells rather than osteoblasts in relationship with multiple myeloma bone disease.. Exp Hematol.

[9] Capdeville R, Buchdunger E, Zimmermann J, Matter A (2002). Glivec (STI571, imatinib), a rationally developed, targeted anticancer drug.. Nat Rev Drug Discov.

[10] Karaman MW, Herrgard S, Treiber DK, Gallant P, Atteridge CE (2008). A quantitative analysis of kinase inhibitor selectivity.. Nat Biotechnol.

[11] Fitter S, Dewar AL, Kostakis P, To LB, Hughes TP (2008). Long-term imatinib therapy promotes bone formation in CML patients.. Blood.

[12] Vandyke K, Fitter S, Dewar AL, Hughes TP, Zannettino AC (2010). Dysregulation of bone remodeling by imatinib mesylate.. Blood.

[13] O'Sullivan S, Naot D, Callon K, Porteous F, Horne A (2007). Imatinib promotes osteoblast differentiation by inhibiting PDGFR signaling and inhibits osteoclastogenesis by both direct and stromal cell-dependent mechanisms.. J Bone Miner Res.

[14] Dewar AL, Farrugia AN, Condina MR, Bik To L, Hughes TP (2006). Imatinib as a potential antiresorptive therapy for bone disease.. Blood.

[15] Olivieri A, Manzione L (2007). Dasatinib: a new step in molecular target therapy.. Ann Oncol.

[16] Aguilera DG, Tsimberidou AM (2009). Dasatinib in chronic myeloid leukemia: a review.. Ther Clin Risk Manag.

[17] Vandyke K, Dewar AL, Farrugia AN, Fitter S, Bik To L (2009). Therapeutic concentrations of dasatinib inhibit in vitro osteoclastogenesis.. Leukemia.

[18] Brownlow N, Mol C, Hayford C, Ghaem-Maghami S, Dibb NJ (2009). Dasatinib is a potent inhibitor of tumour-associated macrophages, osteoclasts and the FMS receptor.. Leukemia.

[19] Lee YC, Huang CF, Murshed M, Chu K, Araujo JC (2010). Src family kinase/abl inhibitor dasatinib suppresses proliferation and enhances differentiation of osteoblasts.. Oncogene.

[20] Id Boufker H, Lagneaux L, Najar M, Piccart M, Ghanem G (2010). The Src inhibitor dasatinib accelerates the differentiation of human bone marrow-derived mesenchymal stromal cells into osteoblasts.. BMC Cancer.

[21] Tibullo D, Barbagallo I, Giallongo C, La Cava P, Branca A (2011). Effects of second-generation tyrosine kinase inhibitors towards osteogenic differentiation of human mesenchymal cells of healthy donors..

[22] Jonsson S, Hjorth-Hansen H, Olsson B, Wadenvik H, Sundan A (2010). Second-generation TKI dasatinib inhibits proliferation of mesenchymal stem cells and osteoblast differentiation in vitro.. Leukemia.

[23] Mihara K, Imai C, Coustan-Smith E, Dome JS, Dominici M (2003). Development and functional characterization of human bone marrow mesenchymal cells immortalized by enforced expression of telomerase.. Br J Haematol.

[24] Wang L, Zhao G, Olivares-Navarrete R, Bell BF, Wieland M (2006). Integrin beta1 silencing in osteoblasts alters substrate-dependent responses to 1,25-dihydroxy vitamin D3.. Biomaterials.

[25] Garayoa M, Garcia JL, Santamaria C, Garcia-Gomez A, Blanco JF (2009). Mesenchymal stem cells from multiple myeloma patients display distinct genomic profile as compared with those from normal donors.. Leukemia.

[26] Dominici M, Le Blanc K, Mueller I, Slaper-Cortenbach I, Marini F (2006). Minimal criteria for defining multipotent mesenchymal stromal cells. The International Society for Cellular Therapy position statement.. Cytotherapy.

[27] Maiso P, Carvajal-Vergara X, Ocio EM, Lopez-Perez R, Mateo G (2006). The histone deacetylase inhibitor LBH589 is a potent antimyeloma agent that overcomes drug resistance.. Cancer Res.

[28] Gregory CA, Gunn WG, Peister A, Prockop DJ (2004). An Alizarin red-based assay of mineralization by adherent cells in culture: comparison with cetylpyridinium chloride extraction.. Anal Biochem.

[29] Xiao G, Jiang D, Ge C, Zhao Z, Lai Y (2005). Cooperative interactions between activating transcription factor 4 and Runx2/Cbfa1 stimulate osteoblast-specific osteocalcin gene expression.. J Biol Chem.

[30] Susa M, Luong-Nguyen NH, Cappellen D, Zamurovic N, Gamse R (2004). Human primary osteoclasts: in vitro generation and applications as pharmacological and clinical assay.. J Transl Med.

[31] Chaudhary LR, Hofmeister AM, Hruska KA (2004). Differential growth factor control of bone formation through osteoprogenitor differentiation.. Bone.

[32] Hock JM, Canalis E (1994). Platelet-derived growth factor enhances bone cell replication, but not differentiated function of osteoblasts.. Endocrinology.

[33] Tokunaga A, Oya T, Ishii Y, Motomura H, Nakamura C (2008). PDGF receptor beta is a potent regulator of mesenchymal stromal cell function.. J Bone Miner Res.

[34] Marzia M, Sims NA, Voit S, Migliaccio S, Taranta A (2000). Decreased c-Src expression enhances osteoblast differentiation and bone formation.. J Cell Biol.

[35] Veracini L, Franco M, Boureux A, Simon V, Roche S (2005). Two functionally distinct pools of Src kinases for PDGF receptor signalling.. Biochem Soc Trans.

[36] Bantscheff M, Eberhard D, Abraham Y, Bastuck S, Boesche M (2007). Quantitative chemical proteomics reveals mechanisms of action of clinical ABL kinase inhibitors.. Nat Biotechnol.

[37] Kratchmarova I, Blagoev B, Haack-Sorensen M, Kassem M, Mann M (2005). Mechanism of divergent growth factor effects in mesenchymal stem cell differentiation.. Science.

[38] Wang X, Goh CH, Li B (2007). p38 mitogen-activated protein kinase regulates osteoblast differentiation through osterix.. Endocrinology.

[39] Choi YH, Gu YM, Oh JW, Lee KY (2011). Osterix is regulated by Erk1/2 during osteoblast differentiation.. Biochem Biophys Res Commun.

[40] Kulterer B, Friedl G, Jandrositz A, Sanchez-Cabo F, Prokesch A (2007). Gene expression profiling of human mesenchymal stem cells derived from bone marrow during expansion and osteoblast differentiation.. BMC Genomics.

[41] Clevers H (2006). Wnt/beta-catenin signaling in development and disease.. Cell.

[42] Qiang YW, Hu B, Chen Y, Zhong Y, Shi B (2009). Bortezomib induces osteoblast differentiation via Wnt-independent activation of beta-catenin/TCF signaling.. Blood.

[43] Vaananen HK, Laitala-Leinonen T (2008). Osteoclast lineage and function.. Arch Biochem Biophys.

[44] Teitelbaum SL (2000). Bone resorption by osteoclasts.. Science.

[45] Asagiri M, Takayanagi H (2007). The molecular understanding of osteoclast differentiation.. Bone.

[46] Grigoriadis AE, Wang ZQ, Cecchini MG, Hofstetter W, Felix R (1994). c-Fos: a key regulator of osteoclast-macrophage lineage determination and bone remodeling.. Science.

[47] Wang ZQ, Ovitt C, Grigoriadis AE, Mohle-Steinlein U, Ruther U (1992). Bone and haematopoietic defects in mice lacking c-fos.. Nature.

[48] Takayanagi H (2007). The role of NFAT in osteoclast formation.. Ann N Y Acad Sci.

[49] Martinez-Martinez S, Rodriguez A, Lopez-Maderuelo MD, Ortega-Perez I, Vazquez J (2006). Blockade of NFAT activation by the second calcineurin binding site.. J Biol Chem.

[50] Nakamura I, Duong le T, Rodan SB, Rodan GA (2007). Involvement of alpha(v)beta3 integrins in osteoclast function.. J Bone Miner Metab.

[51] Han JH, Choi SJ, Kurihara N, Koide M, Oba Y (2001). Macrophage inflammatory protein-1alpha is an osteoclastogenic factor in myeloma that is independent of receptor activator of nuclear factor kappaB ligand.. Blood.

[52] Lentzsch S, Gries M, Janz M, Bargou R, Dorken B (2003). Macrophage inflammatory protein 1-alpha (MIP-1 alpha ) triggers migration and signaling cascades mediating survival and proliferation in multiple myeloma (MM) cells.. Blood.

[53] Roodman GD, Dougall WC (2008). RANK ligand as a therapeutic target for bone metastases and multiple myeloma.. Cancer Treat Rev.

[54] Charbord P (2010). Bone marrow mesenchymal stem cells: historical overview and concepts.. Hum Gene Ther.

[55] Pittenger MF, Mackay AM, Beck SC, Jaiswal RK, Douglas R (1999). Multilineage potential of adult human mesenchymal stem cells.. Science.

[56] Kagami H, Agata H, Tojo A (2011). Bone marrow stromal cells (bone marrow-derived multipotent mesenchymal stromal cells) for bone tissue engineering: basic science to clinical translation.. Int J Biochem Cell Biol.

[57] Khosla S, Westendorf JJ, Modder UI (2010). Concise review: Insights from normal bone remodeling and stem cell-based therapies for bone repair.. Stem Cells.

[58] Nandi SK, Roy S, Mukherjee P, Kundu B, De DK (2010). Orthopaedic applications of bone graft & graft substitutes: a review.. Indian J Med Res.

[59] Mazo M, Gavira JJ, Abizanda G, Moreno C, Ecay M (2010). Transplantation of mesenchymal stem cells exerts a greater long-term effect than bone marrow mononuclear cells in a chronic myocardial infarction model in rat.. Cell Transplant.

[60] Luo FR, Yang Z, Camuso A, Smykla R, McGlinchey K (2006). Dasatinib (BMS-354825) pharmacokinetics and pharmacodynamic biomarkers in animal models predict optimal clinical exposure.. Clin Cancer Res.

[61] Vandyke K, Dewar AL, Diamond P, Fitter S, Schultz CG (2010). The tyrosine kinase inhibitor dasatinib dysregulates bone remodelling through inhibition of osteoclasts in vivo.. J Bone Miner Res.

[62] Christopher LJ, Cui D, Wu C, Luo R, Manning JA (2008). Metabolism and disposition of dasatinib after oral administration to humans.. Drug Metab Dispos.

[63] Sambrook P, Cooper C (2006). Osteoporosis.. Lancet.

[64] Voorzanger-Rousselot N, Goehrig D, Journe F, Doriath V, Body JJ (2007). Increased Dickkopf-1 expression in breast cancer bone metastases.. Br J Cancer.

[65] Roodman GD (2009). Pathogenesis of myeloma bone disease.. Leukemia.

[66] Garderet L, Mazurier C, Chapel A, Ernou I, Boutin L (2007). Mesenchymal stem cell abnormalities in patients with multiple myeloma.. Leuk Lymphoma.

[67] Wallace SR, Oken MM, Lunetta KL, Panoskaltsis-Mortari A, Masellis AM (2001). Abnormalities of bone marrow mesenchymal cells in multiple myeloma patients.. Cancer.

[68] Yaccoby S (2010). Osteoblastogenesis and tumor growth in myeloma.. Leuk Lymphoma.

[69] Giuliani N, Rizzoli V, Roodman GD (2006). Multiple myeloma bone disease: Pathophysiology of osteoblast inhibition.. Blood.

[70] Coluccia AM, Cirulli T, Neri P, Mangieri D, Colanardi MC (2008). Validation of PDGFRbeta and c-Src tyrosine kinases as tumor/vessel targets in patients with multiple myeloma: preclinical efficacy of the novel, orally available inhibitor dasatinib.. Blood.

[71] Rosen LS (2004). New generation of bisphosphonates: broad clinical utility in breast and prostate cancer.. Oncology (Williston Park).

[72] Terpos E, Sezer O, Croucher PI, Garcia-Sanz R, Boccadoro M (2009). The use of bisphosphonates in multiple myeloma: recommendations of an expert panel on behalf of the European Myeloma Network.. Ann Oncol.

